# Supplementary material for: miR-34a is a microRNA safeguard for Citrobacter-induced inflammatory colon oncogenesis
Source: eLife. 2018 Dec 13;7:e39479. doi: 10.7554/eLife.39479 (PMC6314783; doi:10.7554/eLife.39479)
Supplement: Figure 7—source data 1. — This file contains the information of CRC patients. [file elife-39479-fig7-data1.doc]

**Figure 7-source data 1. Source data for Figure 7. This file contains the information of CRC patients.**

| **Patient** | **Gender** | **Age at visit** | **Stage** | **Differentiation** | **Lymph nodes** |
| --- | --- | --- | --- | --- | --- |
| P1 | F | 59**.56** | IIB | Poor | N0 |
| P2 | F | 73 | IV | Moderate | N1-3 |
| P3 | M | 73 | IIIA | Well | N1-3 |
| P4 | M | 56 | IV | Poor | N2>3 |
| P5 | F | 61 | IIIC | Poor | N2>3 |
| P6 | M | 87 | IIB | NA | N0 |
| P7 | F | 74 | IIIA | Poor | N1-3 |
| P8 | F | 68 | IIA | Poor | N0 |
| P9 | F | 62 | IIIB | Poor | N1-3 |
| P10 | F | 77 | IIIB | Poor | N1-3 |
| P11 | F | 46 | IV | - | N1-3 |
| P12 | M | 54 | I | Moderately | N0 |
| P13 | M | 85 | IIIC | Moderately | N2>3 |
| P14 | M | 79 | 3A | Poor | N1-3 |
| P15 | F | 92 | 2B | Well | N0 |
| P16 | F | 81 | 2A | Moderately | N0 |
| P17 | F | 84 | 1 | Moderately | N0 |
