# Supplementary material for: miR-34a is a microRNA safeguard for Citrobacter-induced inflammatory colon oncogenesis
Source: eLife. 2018 Dec 13;7:e39479. doi: 10.7554/eLife.39479 (PMC6314783; doi:10.7554/eLife.39479)
Supplement: Figure 7—source data 2. — This file contains RT-qPCR primers used in this study. [file elife-39479-fig7-data2.doc]

| **Primer name** | **Sequence** |
| --- | --- |
| mIL-4F | ACTTGAGAGAGATCATCGGCA |
| mIL-4R | AGCTCCATGAGAACACTAGAGTT |
| mIL-17F | CTTTCCCTCCGCATTGACAC |
| mIL-17R | TTTAACTCCCTTGGCGCAAAA |
| mIFN-γ-F | TCA AGT GGC ATA GAT GTG GAA GAA |
| mIFN-γ-F | TGG CTC TGC AGG ATT TTC ATG |
| mFoxp3-F | CCCAGGAAAGACAGCAACCTT |
| mFoxp3-R | TTCTCACAACCAGGCCACTTG |
| mCCR4-F | AACAGAGCAGTGCGCATGAT |
| mCCR4-R | CGTTGTACGGCGTCCAGAA |
| mCCR6-F | CCTCACATTCTTAGGACTGGAGC |
| mCCR6-R | GGCAATCAGAGCT CTCGGA |
| mCCL20-F | ATGGCCTGCGGTGGCAAGCGTCTG |
| mCCL20-R | TAGGCTGAGGAGGTTCACAGCCCT |
| mCCL22-F | GTG GCT CTC GTC CTT CTT GC |
| mCCL22-R | GGA CAG TTT ATG GAG TAG CTT |
| mIL-17RA-F | AGTGTTTCCTCTACCCAGCAC |
| mIL-17RA-R | GAAAACCGCCACCGCTTAC |
| mIL-17RC-F | GGAGCAGGACTTTAGCTTCTT |
| mIL-17RC-R | GAACCAGGTCTGTGTGGTTTA |
| mIL-17RD-F | TGATGCAATCAAGAGCCAGACAG |
| mIL-17RD-R | GGTAGCCACACAGGGCCAATA |
| mactin-F | GGCTGTATTCCCCTCCATCG |
| mactin-R | CCAGTTGGTAACAATGCCATGT |
| hIL-17F | TCAACCCGATTGTCCACCAT |
| hIL-17R | GAGTTTAGTCCGAAATGAGGCTG |
| hRORC-F | GAAGTGGTGCTGGTTAGGATGTG |
| hRORC-R | GCCACCGTATTTGCCTTCAA |
| hactin -F | CGCGAGAAGATGACCCAGAT |
| hactin -R | ACAGCCTGGATAGCAACGTACAT |

**Figure 7-source data 4. Source data for Figure 7. This file contains RT-qPCR primers used in this study.**
